# Multilayered Plasmonic Heterostructure of Gold and Titania Nanoparticles for Solar Fuel Production

**DOI:** 10.1038/s41598-018-28789-w

**Published:** 2018-07-11

**Authors:** Jeonga Kim, Ho Yeon Son, Yoon Sung Nam

**Affiliations:** 1 Department of Materials Science and Engineering, Korea Advanced Institute of Science and Technology, 291 Daehak-ro, Yuseong-gu, Daejeon 34141 Republic of Korea; 2 KAIST Institute for the NanoCentury, Korea Advanced Institute of Science and Technology, 291 Daehak-ro, Yuseong-gu, Daejeon 34141 Republic of Korea

## Abstract

Solar fuel production via photoelectrochemical (PEC) water splitting has attracted great attention as an approach to storing solar energy. However, a wide range of light-harvesting materials is unstable when exposed to light and oxidative conditions. Here we report a robust, multilayered plasmonic heterostructure for water oxidation using gold nanoparticles (AuNPs) as light-harvesting materials via localized surface plasmon resonance (LSPR). The multilayered heterostructure is fabricated using layer-by-layer self-assembly of AuNPs and TiO_2_ nanoparticles (TNPs). Plasmon-induced hot electrons are transferred from AuNPs to TNPs over the Au/TiO_2_ Schottky barrier, resulting in charge separation of hot carriers. Plasmonic photoanodes for water oxidation are completed by incorporating a Co-based oxygen-evolving catalyst on the multilayered heterostructure to scavenge hot holes. Light absorption capability and PEC properties of the photoanodes are investigated as a function of the number of AuNP/TNP bilayers. The PEC properties exhibits dependence on the number of the bilayers, which is affected by charge transport within the multilayered heterostructures. Photocurrent density and decrease in impedance by irradiation indicates significant photoactivity by LSPR excitation.

## Introduction

Solar-to-hydrogen conversion can be driven through photoelectrochemical (PEC) water splitting using water as an electron source and harnessing photopotential from solar energy^1^. Photochemical capabilities of materials such as light absorption, light-induced charge carrier generation, charge separation, and charge transfer have been emphasized to be used as a photoelectrode^2–5^. The photochemical stability of materials is also very important because the oxidative environment of water oxidation induces the corrosion and decomposition of photoelectrodes in contact with aqueous electrolytes under light illumination^6,7^. Wide band gap semiconductors (e.g., TiO_2_, WO_3_, and SrTiO_3_) have been widely used because of their excellent stability in the oxidative environment, but they can absorb only the UV light, which accounts for about 5% in the solar irradiance spectrum, resulting in a low solar-to-hydrogen conversion efficiency^8–10^. Narrow band gap semiconductors (e.g., Si, CdS, and CdSe) can cover a broader solar spectrum, but they are very unstable due to photocorrosion^7,8,11^. Molecular photosensitizers (e.g., ruthenium complexes and porphyrins) have been studied due to their structural and spectral tunability^12,13^. However, they also degrade very quickly when exposed to light^14–17^. The instability of the materials makes wide band gap semiconductors preferentially chosen for a photoanode, and many efforts have been made to overcome their limited photoactivity in the visible light region through controlled doping and oxygen vacancy^18–21^.

Recently, plasmonic metals have attracted increasing attention as a light-harvesting material for PEC and photocatalytic applications because they are stable and strongly respond to visible light via localized surface plasmon resonance (LSPR) with excellent absorption cross-section^22^. After excitation, the LSPR energy decays non-radiatively via Landau damping, creating highly energetic charge carriers, which are energetic electron-hole pairs, denoted as ‘hot carriers’^23^. Plasmon-induced hot carriers decay through electron-electron (10–100 fs) and electron-phonon (100 fs–1 ps) relaxations to cause thermal dissipation of the LSPR energy^22,24,25^. This ultrafast relaxation makes it crucial for the plasmon-induced hot carriers to be rapidly separated and transferred to drive a chemical reaction^26^. Heterostructures of a plasmonic metal nanostructure and a semiconductor have been utilized to harness plasmon-induced hot carriers by efficiently separating the hot carriers through the Schottky barrier^27–31^. The Schottky barrier can be formed at a metal/semiconductor interface when metal is in contact with the semiconductor with an appropriate electronic structure, facilitating charge separation and increasing a lifetime of the hot carriers^32,33^. Hot electrons having the energy higher than the Schottky barrier can be injected from the plasmonic metal into the conduction band (CB) of the semiconductor.

Recent works demonstrated that plasmonic metal could serve as a photosensitizer for visible light, and hot electron injection over the Schottky barrier could enhance photocatalytic performances^7,32,34–36^. However, no previous reports have attempted to increase the interfacial area of plasmonic metal and semiconductor nanostructures and investigate its impact on the separation and collection of the hot carriers for PEC water splitting. Thus, a new system that can efficiently separate and collect plasmon-induced hot carriers needs to be developed to enhance PEC water splitting. In addition, fast scavenging of hot holes from the plasmonic metals is required for efficient plasmon-induced charge separation due to the ultrafast decay of plasmon-induced hot carriers. Although the use of catalysts is important for the fast extraction of hot holes, only a few works reported the incorporation of water oxidation catalysts into plasmonic photocatalytic systems^7,34,37^.

**Figure 1:**
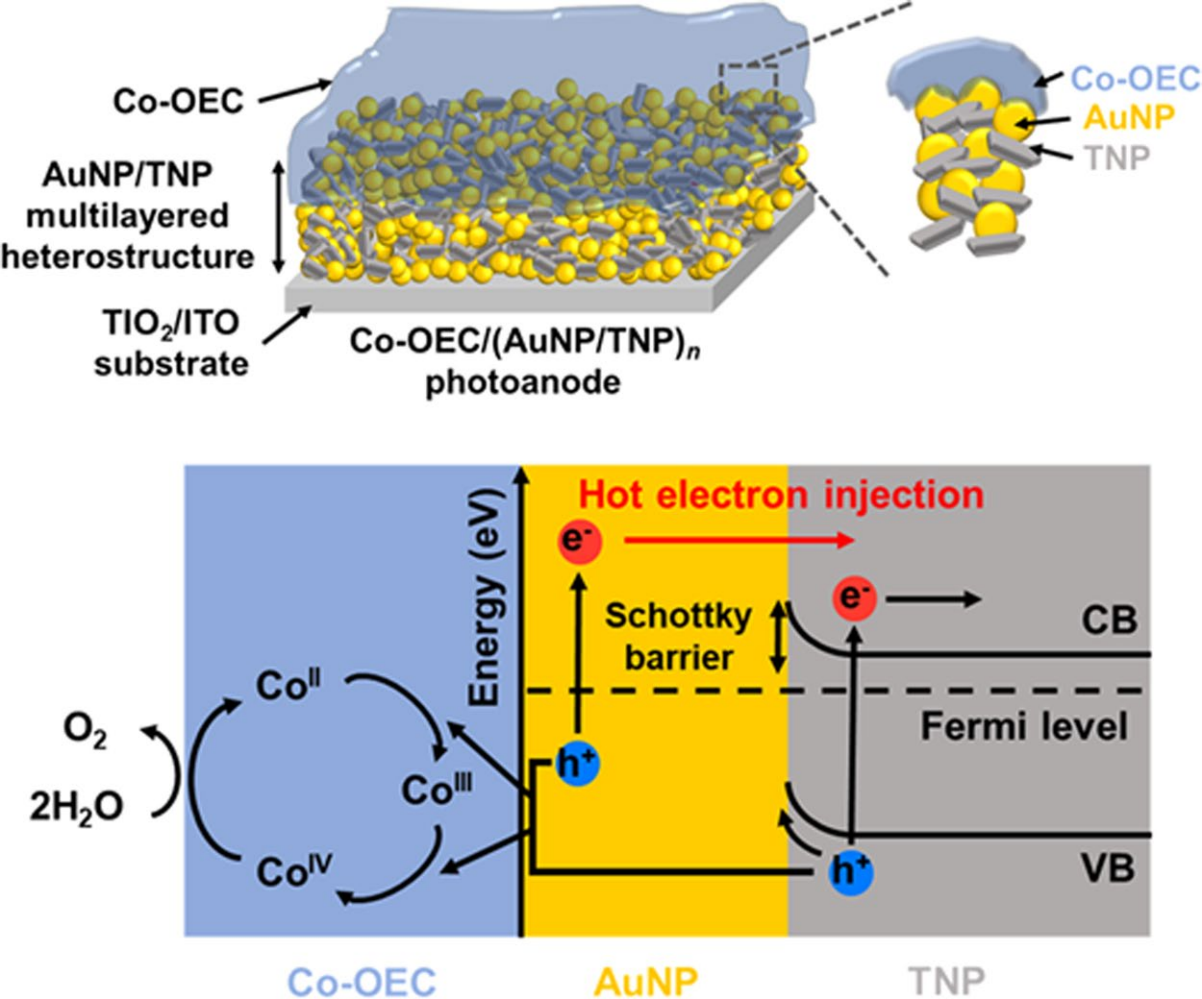
Schematic illustration of the working principle of the Co-OEC/(AuNP/TNP)_*n*_ photoanode.

In this report, we introduce a plasmonic catalytic photoanode based on a multilayered heterostructure containing gold nanoparticles (AuNPs) and anatase TiO_2_ nanoparticles (TNPs) with a cobalt-based oxygen evolution catalyst (Co-OEC). A layer-by-layer (LbL) self-assembly technique was employed to prepare the multilayered plasmonic heterostructure, aimed to increase the number of the hot electrons being injected into the semiconductor through a large interfacial area. The plasmonic photoanode was completed by incorporating the Co-OEC onto the multilayered heterostructure. The Co-OEC can improve hot hole transfer into electrolyte by accelerating the kinetics of water oxidation, which facilitates the charge separation of the hot carriers and allows the efficient utilization of plasmon-induced hot carriers. Working principles of the photoanode proposed in the present study are described in Fig. 1. The hot electrons move to the CB of TiO_2_ across the Schottky barrier, and photoexcited electrons from valence band (VB) to CB in the TiO_2_ contribute to photocurrent when irradiated by solar light, finally driving the reduction reaction. Meanwhile, hot holes are transferred from AuNPs to the interface of Co-OEC, which drives the water oxidation reaction. The present work investigated the optical, structural, and photoelectrochemical properties of the photoanodes with different AuNP/TNP bilayer numbers to examine their applicability to PEC water splitting.

## Results and Discussion

### Synthesis and Characterization of Positively Charged AuNPs and Negatively Charged TNPs

**Figure 2:**
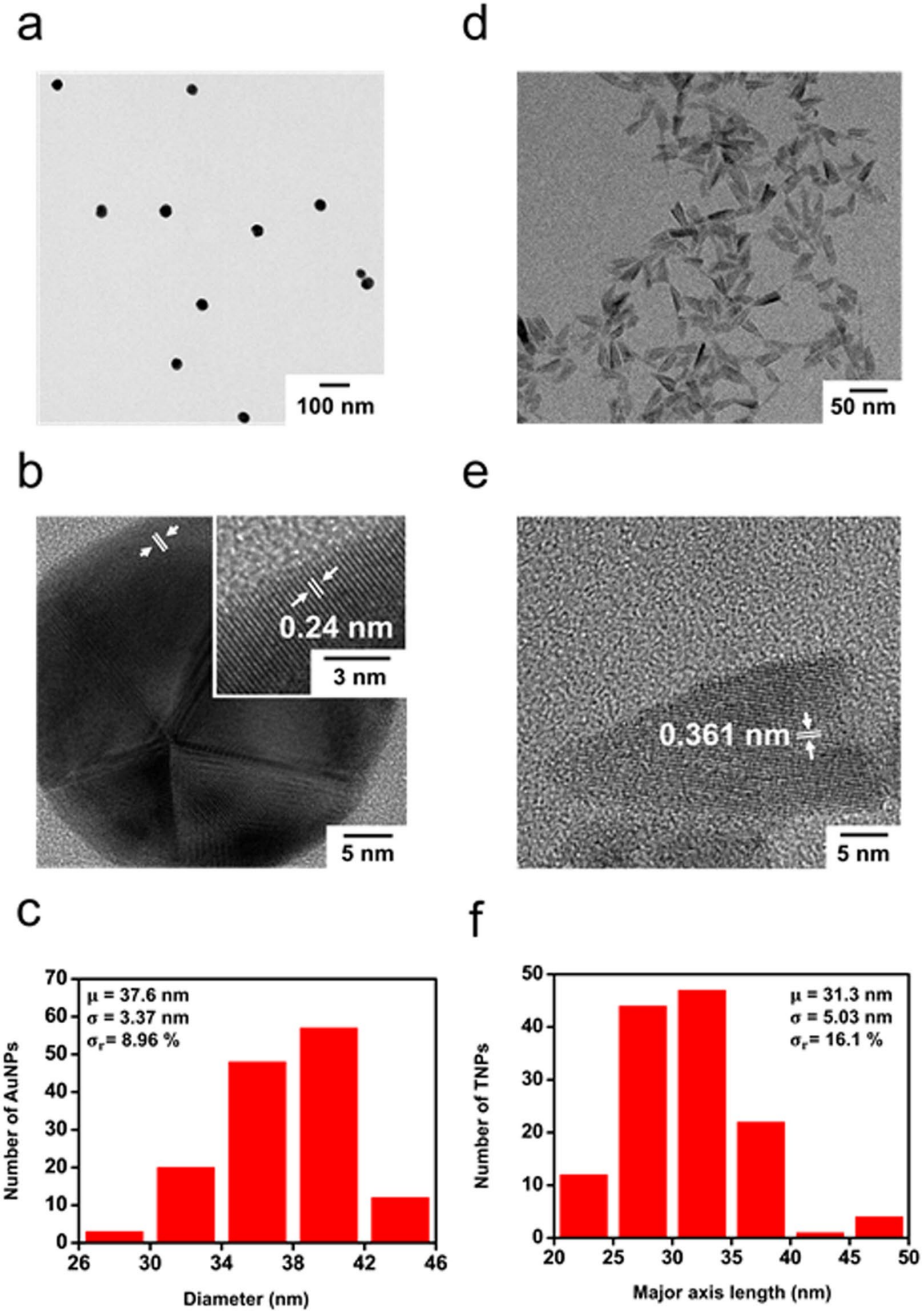
TEM images and particle size distribution histograms of the synthesized AuNPs (**a**–**c**) (b, inset: high-resolution TEM image for AuNPs) and TNPs (**d**–**f**).

Positively charged AuNPs were synthesized using sodium borohydride (NaBH_4_) and cysteamine as reducing and capping agents, respectively. The primary amine group of cysteamine was protonated and positively charged during the synthesis of AuNPs at a neutral pH due to its high pK_a_ value (10.75)^38^. The synthesized AuNPs were well dispersed in deionized water, and their maximum absorption peak appeared at 526 nm due to the LSPR of AuNPs (Supplementary Fig. S1a,b). The synthesized AuNPs had spherical or ellipsoidal shapes (Fig. 2a). The AuNPs had a polycrystalline phase with a lattice fringe spacing of 0.24 nm, which corresponds to the (111) plane of face-centered cubic Au, as shown in a high-resolution transmission electron microscope (TEM) image (Fig. 2b)^39^. The average diameter of AuNPs was 37.6 ± 3.37 nm, as measured from 140 particles from TEM images (Fig. 2c).

Negatively charged anatase TNPs were prepared by the hydrolysis and condensation of titanium (IV) isopropoxide (TTIP) using tetrabutylammonium hydroxide 30-hydrate (TBAH) as a base^40,41^. Due to the surface hydroxyl groups, the colloidal TNPs have an isoelectric point between 4.7 and 6.2^42^. The absorption intensity of TNPs was sharply increased below 400 nm due to the interband transition, and the maximum absorption was observed at 242 nm, as shown in Supplementary Fig. S2. The negatively charged TNPs had a short rod-like shape with average major and minor axes lengths of 31.3 ± 5.03 nm and 11.9 ± 2.08 nm, respectively (Fig. 2d–f and Supplementary Fig. S3). Specifically, TNPs have arrowhead shapes dominantly with some ellipsoidal shapes owing to the different growth rates in the [101] and [001] directions by TBAH. The high-resolution TEM exhibited a single-crystalline phase with a lattice fringe spacing of 0.361 nm, which corresponds to the (101) plane of anatase titania (Fig. 2e)^43^. The average zeta potentials were 36.17 mV and −34.65 mV for AuNPs and TNPs, respectively, indicating that AuNPs and TNPs are positively and negatively charged as expected.

### LbL Self-assembly of AuNPs and TNPs

**Figure 3:**
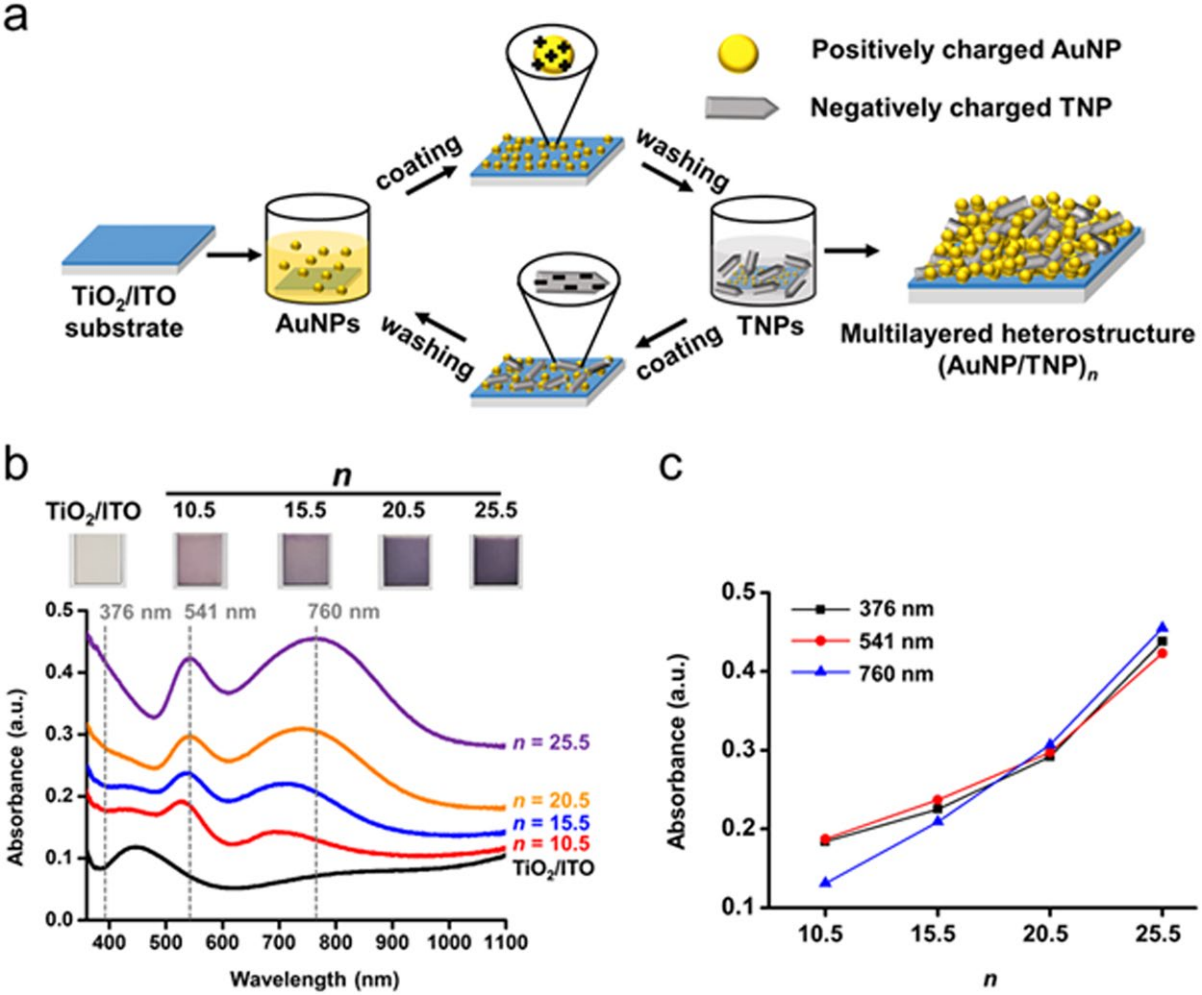
(**a**) Schematic illustration of the fabrication procedures of (AuNP/TNP)_*n*_ multilayered heterostructures. Absorption spectra and digital photograph (**b**), and absorbance vs. *n* plot at specific wavelengths (**c**) for (AuNP/TNP)_*n*_ multilayered heterostructures.

Multilayered plasmonic heterostructures were fabricated by the LbL self-assembly of AuNPs and TNPs via electrostatic interactions, as schematically described in Fig. 3a. LbL self-assembly was performed by immersing a substrate in the colloidal solutions of AuNPs and TNPs alternatively. Washing steps were added between the immersion steps to remove unbound nanoparticles. AuNPs and TNPs were used as a visible light photosensitizer and a hot electron filter, respectively. In the multilayered heterostructure, a metal/semiconductor Schottky contact is generated at the interface between AuNPs and TNPs, enabling the efficient hot electron injection from AuNPs to TNPs. A TiO_2_ thin film was deposited on the indium-doped tin oxide (ITO)-coated glass slide by e-beam evaporation and used as a substrate for the fabrication of the multilayered heterostructures (denoted as ‘TiO_2_/ITO’). The surface of TiO_2_/ITO was treated by oxygen plasma before the LbL self-assembly. AuNPs were coated as a top layer to use hot holes generated in AuNPs for an oxidation reaction at the electrode/electrolyte interface. In this work, the multilayered coatings of AuNP and TNPs were conducted with the *n* numbers of 10.5, 15.5, 20.5, and 25.5, indicating the number of layers (denoted as ‘(AuNP/TNP)_*n*_’). For example, the *n* number of 10.5 indicates that the photoanode has ten AuNP/TNP bilayers and a top AuNP layer.

### Characterization of (AuNP/TNP)_*n*_ Multilayered Heterostructures

Digital photographs and light absorption spectra of (AuNP/TNP)_*n*_ exhibit the dependency of the color of the active area in the photoanode on the number of AuNP/TNP bilayers (Fig. 3b). The color of the active area became darker violet with increasing *n* from 10.5 to 25.5 due to the increased amounts of AuNPs deposited on the electrode. The absorbance in the whole range of wavelengths was increased with the increased number of the bilayers. The absorption in the visible light and near-infrared (NIR) regions was raised by the increased number of AuNPs, while the increased absorption in the UV region was attributed to the increased number of TNPs. Specifically, the absorption spectra exhibited three absorption bands: one due to interband transition in TNPs in the range from 370 to 390 nm, another due to the LSPR of isolated and individual AuNPs around 540 nm, and the other due to plasmonic coupling at assemblies of AuNPs in the range from 680 to 750 nm. The absorption intensities at 376, 541, and 760 nm, which represent each spectral region, were increased linearly with the increasing number of the bilayers (Fig. 3c). The results indicate that the multilayered heterostructure grew uniformly by repeating the LbL self-assembly, making it easy to adjust the light absorption capability.

The LSPR peak of (AuNP/TNP)_*n*_ was red-shifted with respect to that of the colloidal AuNPs presumably due to the change in the dielectric constant of surrounding media and the plasmonic coupling of AuNPs during the LbL self-assembly. A resonant condition in the LSPR depends on the dielectric constant of a medium surrounding the plasmonic nanostructures^44^. AuNPs in a colloidal solution are surrounded by water (dielectric constant *ε*_*r*_ = 1.78) and cysteamines (refractive index ~ 1.5), while AuNPs on the photoanodes are mostly surrounded by anatase TNPs (ε_r_ = 31) and air (*ε*_*r*_ ~ 1)^5,45,46^. The increased dielectric constants of surrounding media lead to the red-shift of the resonant wavelength of AuNPs. The absorption peaks of (AuNP/TNP)_*n*_ at the larger wavelengths were caused by the assemblies of AuNPs. The assemblies of AuNPs lead to strong light absorption in a lower energy region (e.g., NIR region) because of plasmonic coupling between adjacent AuNPs, which can produce the nearly violet or black with the increased number of AuNP layers, as shown in the photograph^47,48^.

**Figure 4:**
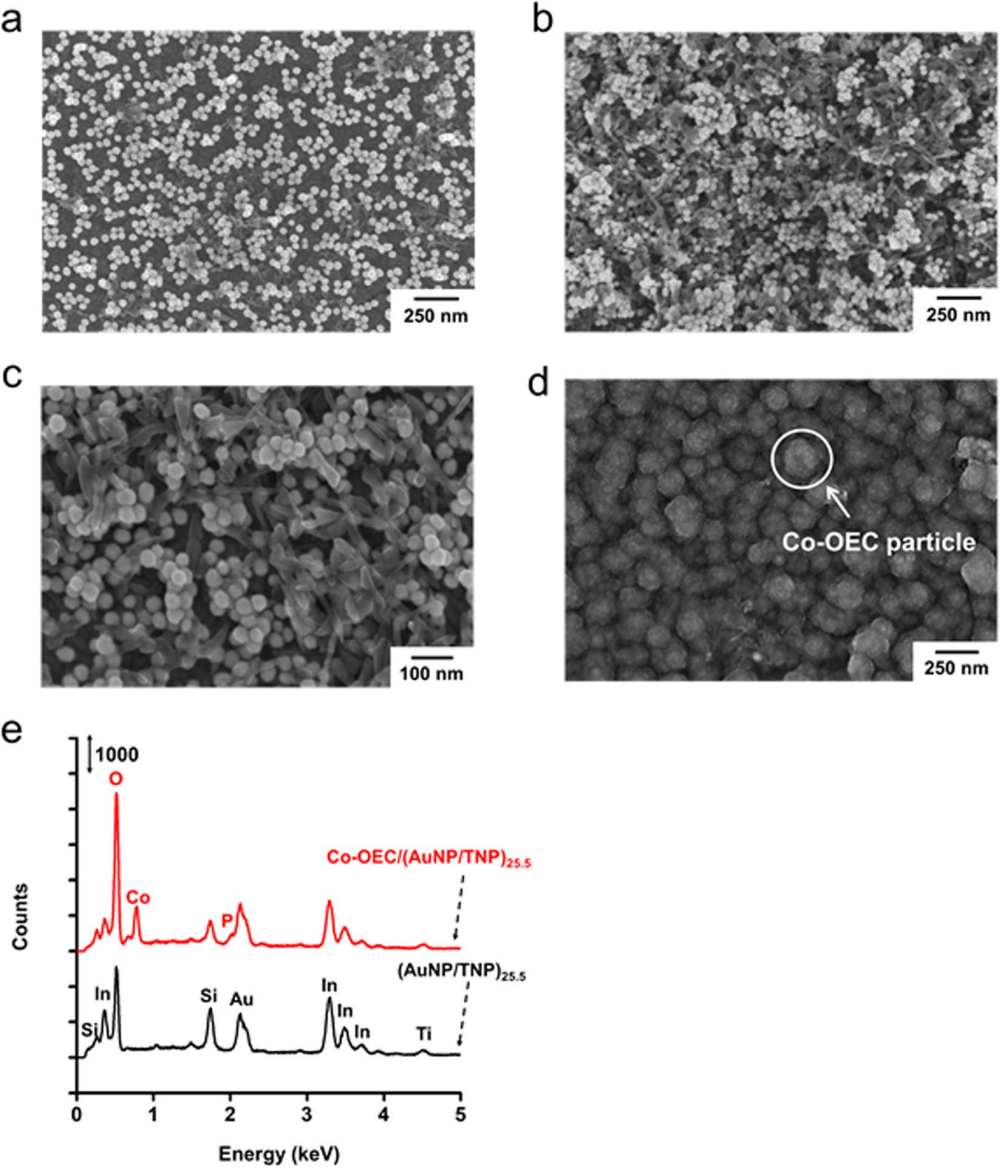
SEM images of the (AuNP/TNP)_*n*_ multilayered heterostructure with *n* = 10.5 (**a**) and 25.5 (**b** and **c**) and the Co-OEC/(AuNP/TNP)_*n*_ photoanode with *n* = 25.5 (**d**). EDAX spectra (**e**) of *n* = 25.5 with or without Co-OEC.

Scanning electron microscope (SEM) images of the (AuNP/TNP)_*n*_ show AuNPs and TNPs on the substrate as brighter spherical dots and darker facets, respectively (Fig. 4a–c and Supplementary Fig. S4). The number density of nanoparticles was increased with increasing the number of the bilayers. The formation of multi-composites and interfaces between AuNPs and TNPs was observed (Fig. 4c). The appreciably larger numbers of AuNPs and TNPs were observed at *n* = 25.5 compared to *n* = 10.5, as discussed for the absorption spectra (Fig. 4a,b). The surface coverage of AuNPs and TNPs on the substrate was calculated using the SEM images (Supplementary Fig. S5). The surface coverage depended on *n*, which was increased with a relatively high and constant slope up to *n* = 20.5 and then increased with a lower slope. The results imply that lateral stacking of NPs to a dense structure is dominant up to *n* = 20.5 and then vertical stacking is dominant between *n* = 20.5 and 25.5 during the LbL self-assembly of AuNPs and TNPs.

### Electrodeposition of Co-OEC

Co-OEC was electrodeposited onto the multilayered heterostructures, specifically (AuNP/TNP)_*n*_, using Co^2+^-containing solutions under the presence of potassium phosphate (KPi) buffer (pH 7) at 1.513 V versus a reversible hydrogen electrode (RHE) where a catalytic wave was not observed until 30 mC flowed as reported previously^49^. The electrodeposition potential was determined by comparing the curves measured from the cyclic voltammetry (CV) in KPi electrolyte (pH 7) with and without Co^2+^ ions. Co-OEC-deposited (AuNP/TNP)_*n*_ photoanode is denoted as Co-OEC/(AuNP/TNP)_*n*_. SEM analysis revealed the formation of particle-like structures of Co-OEC on the multilayered heterostructures (Fig. 4d and Supplementary Fig. S6). Energy dispersive X-ray spectroscopy (EDAX) was performed to demonstrate the presence of Co-OEC (Fig. 4e). The EDAX spectrum of Co-OEC/(AuNP/TNP)_25.5_ exhibited signals of Co and P elements and significantly increased the signal of O element, compared to (AuNP/TNP)_25.5_, which confirmed that Co-OEC were formed on the multilayered heterostructures.

### PEC Analysis

PEC analysis for the Co-OEC/(AuNP/TNP)_*n*_ photoanode was conducted to investigate the effects of the number of layers on the charge transfer kinetics. A three-electrode system was used with the photoanode as a working electrode (WE), a Pt mesh as a counter electrode (CE), and Ag/AgCl as a reference electrode (RE). The anode was illuminated through the TiO_2_/ITO substrate in 0.1 M KPi buffer at pH 7. All potentials are expressed with respect to the RHE. To evaluate the catalytic activities of the photoanodes, we measured the PEC properties of the photoanode under AM 1.5 simulated sunlight (denoted as ‘1 sun’), 1 sun passing through a 400 nm shortpass filter (denoted as ‘λ < 400 nm’), 1 sun passing through a 400 nm longpass filter and a 750 nm shortpass filter (denoted as ‘400 nm < λ < 750 nm’), and 1 sun passing through a 750 nm longpass filter (denoted as ‘λ > 750 nm’).

**Figure 5:**
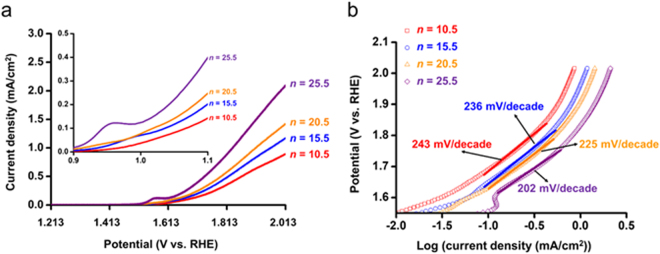
Average LSV curves (**a**) and Tafel plots corresponding to the LSV curves (**b**) for Co-OEC/(AuNP/TNP)_*n*_ photoanodes in the dark.

Linear sweep voltammetry (LSV) was performed in the dark at a scan rate of 2 mV s^−1^ to determine the electrochemical catalytic properties of the photoanodes for *n* = 10.5–25.5, as shown in Fig. 5a. The number of the samples used for LSV measurement is summarized in Supplementary Table S1. The overpotential was reduced for larger *n*, indicating the higher catalytic performance of the thicker multilayered photoanodes. To quantitatively compare the catalytic performance among the photoanodes, Tafel plots were obtained from the LSV curves (Fig. 5b). Tafel slopes for *n* = 10.5, 15.5, 20.5 and 25.5 were 243, 236, 225, and 202 mV decade^−1^ respectively, which also exhibits the more improved catalytic performance for larger *n*. This is possibly because an interfacial area between the multilayered heterostructures and Co-OEC was increased with increasing *n* as a result of the formation of a three-dimensional network of AuNPs and TNPs. Water oxidation reaction can take place throughout Co-OEC, as suggested in previous studies which reported the increased current density with increasing the amount of Co-OEC deposited on a same electrode^50,51^. Thus, current density can be almost the same if both the electrode and the amount of Co-OEC are same. However, although Co-OEC was electrodeposited in the same amount of 30 mC in all of the photoanodes in this study, the process by which electrons are transferred from Co-OEC to the multilayered heterostructures of the photoanodes may vary depending on the number of the layers. As the number of the layers increases, the interfacial area between the multilayered heterostructure and Co-OEC can be raised. As a result, the higher surface area of the multilayered heterostructure can facilitate the acceptance of electrons from Co-OEC.

**Figure 6:**
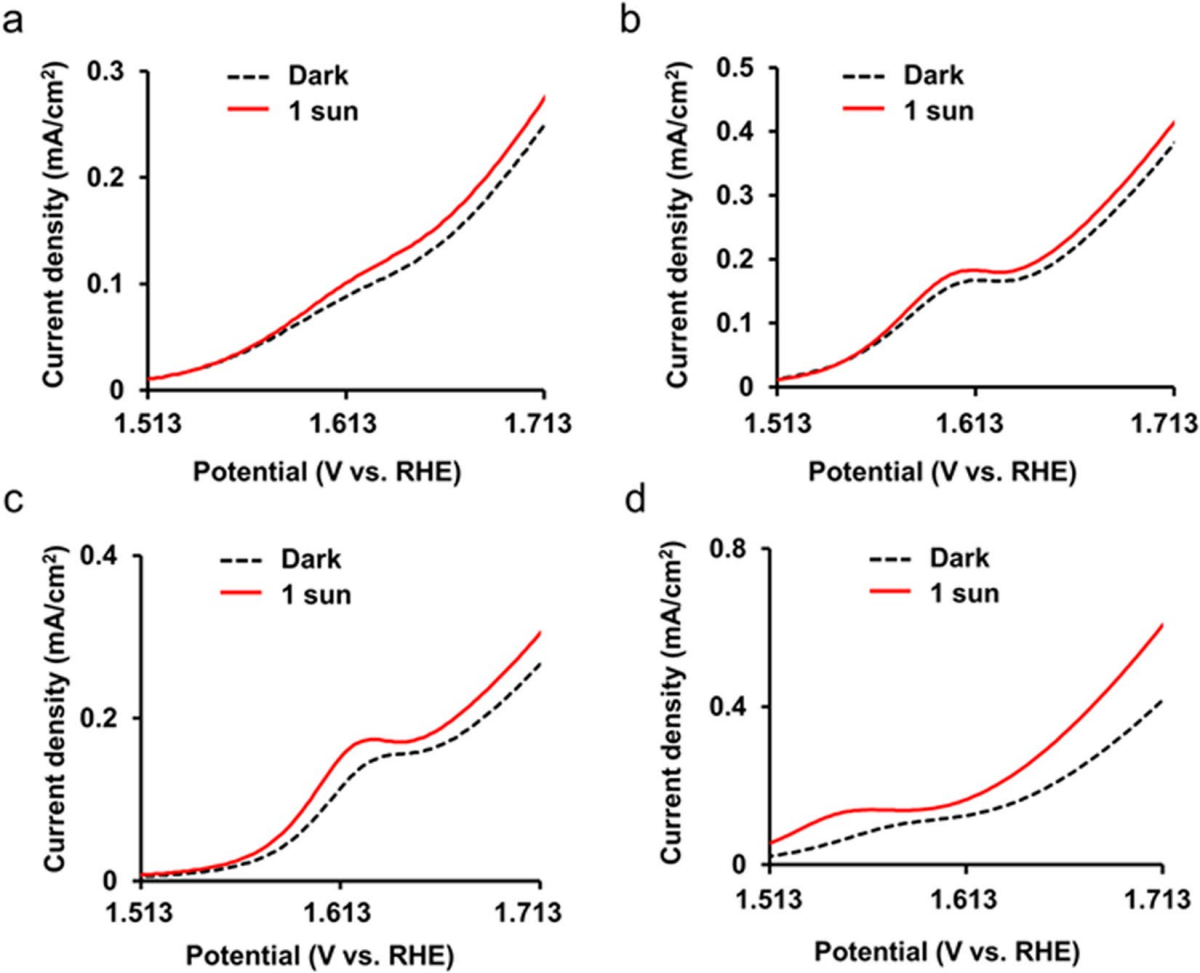
Representative LSV curves under 1 sun and in the dark for Co-OEC/(AuNP/TNP)_*n*_ photoanodes: *n* = 10.5 (**a**), 15.5 (**b**), 20.5 (**c**), and 25.5 (**d**).

The LSV curves of the photoanodes for *n* = 10.5–25.5 showed typical catalytic characteristics of Co-OEC. An anodic wave peak appeared near 1.613 V, which corresponds to the Co^II/III^ redox couple^52^. After Co^II^ was oxidized to Co^III^ near 1.613 V where the anodic wave appeared, a strong catalytic wave was observed at a higher potential than an onset potential due to an oxygen evolution reaction by the Co^IV^-oxo-Co^IV^ clusters formed by Co^III/IV^ oxidation^53,54^. To investigate changes in current density-potential characteristics by irradiation, we conducted LSV at a scan rate of 2 mV s^−1^ under 1 sun and in the dark. Representative LSV curves for each *n* are shown in Fig. 6. The reduced overpotential and increased current density at the same potentials were observed at all of the photoanodes, indicating the existence of photopotential and photocurrent density.

**Figure 7:**
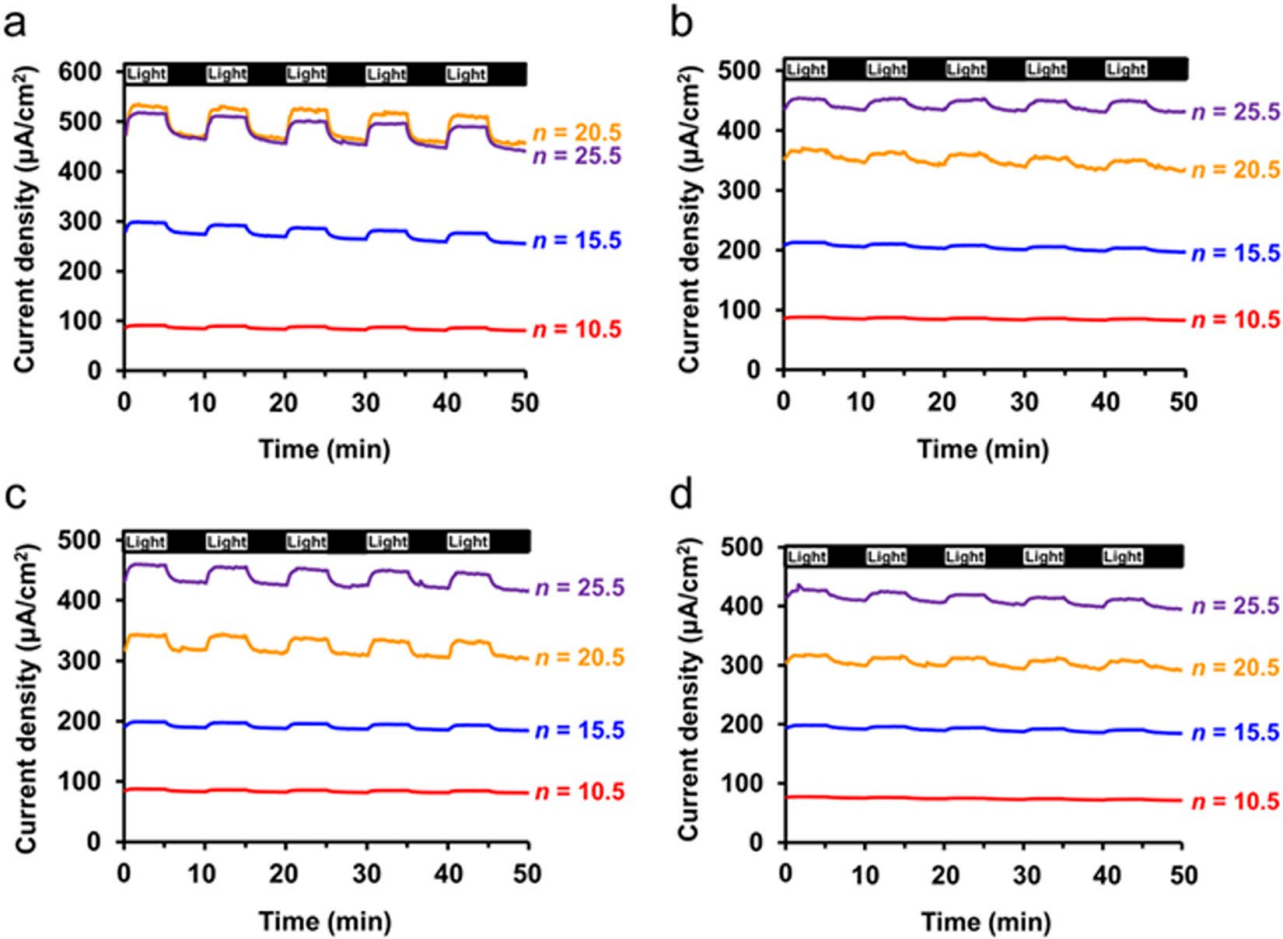
Chronoamperograms at 1.813 V vs. RHE under various chopped illumination conditions: 1 sun (**a**), UV light (λ < 400 nm) (**b**), visible light (400 nm < λ < 750 nm) (**c**), and NIR light (λ > 750 nm) (**d**).

To investigate the photocurrent density, chronoamperometry (CA) analysis was carried out under chopped illumination corresponding to 1 sun at 1.813 V, where oxygen evolution reaction occurs for *n* = 10.5–25.5 due to a higher potential than the onset potential (Fig. 7a). Steady-state photocurrent densities were measured after 5 min of continuous illumination for all the photoanodes. The chronoamperograms revealed that current density was increased rapidly upon illumination and then gradually increased to the steady-state photocurrent density. This increasing transient is due to charge transport in the multilayered heterostructures. According to previous studies, charge transport is mainly affected by an electronic band structure of a bulk part of a photoelectrode, and the electronic band can be slowly bent by photopotential under illumination^55,56^. Band bending within the multilayered heterostructures can be slowly varied, and thereby photocurrent density can be gradually increased to reach the steady-state. In addition, according to Lee *et al*., electrons trapped in TiO_2_ could reduce the Schottky barrier while being transferred from a gold nanorod to TiO_2_, increasing the photocurrent density over time^7^. Similarly, the Schottky barrier can be reduced during the hot electron injection, increasing the photocurrent density. Therefore, the photocurrent density can be determined by charge transport in the photoanode under light illumination at 1.813 V.

To further examine the photoactivity of the photoanodes in the UV, visible, and NIR regions and distinguish the contributions to photocurrent density from each spectral region, chronoamperograms were recorded under UV (λ < 400 nm), visible (400 nm < λ < 750 nm), and NIR light (λ > 750 nm) (Fig. 7b–d). Under various illumination conditions, chronoamperograms also revealed the increasing transient behavior. In the chronoamperograms of *n* = 20.5 and 25.5 at 1.813 V, it was observed that current density values were suddenly bounced and fluctuated in contrast to other smooth chronoamperograms. It seems likely that the water oxidation generated oxygen bubbles at reaction sites of the photoanode and created current density fluctuation during oxygen evolution. Under all the illumination conditions, the photocurrent density was observed. The photocurrent density in the UV region is due to photoexcited electron-hole pairs via the interband transition in TNPs and TiO_2_ thin film where the photoexcited electrons are transported into a back contact of ITO. The reason for the photocurrent density in the visible light region is that hot electron-hole pairs are generated via LSPR in AuNPs and then the hot electrons from AuNPs are injected into TNP or TiO_2_ thin film over the Schottky barrier to enter the back contact.

The reason for the photocurrent density in the NIR region is that the hot electrons generated by NIR light can be transferred over the Schottky barrier to contribute to the current density. However, photon energies of the NIR region are relatively small, so the hot electron injection induced by NIR photons can be limited. In the meantime, the assemblies of AuNPs by plasmonic coupling absorb NIR light and are known to have a large plasmonic heating effect to be utilized in photothermal therapeutic applications^57–59^. The assemblies of AuNPs present in the multilayered heterostructure can generate localized heating at catalytic sites, which can contribute to the catalytic performance and facilitate mass transfer in the electrolyte adjacent to the catalyst.

**Figure 8:**
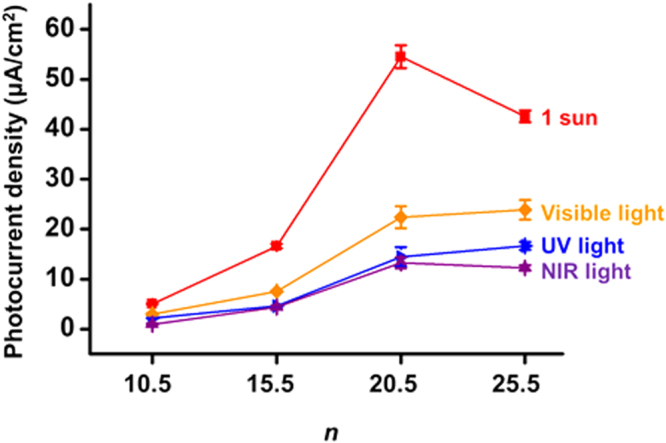
Photocurrent density vs. *n* plots at 1.813 V vs. RHE under various illumination conditions.

Photocurrent densities were calculated from the chronoamperograms to evaluate the photoactivity of the photoanodes. The relationship between the photocurrent density and *n* at 1.813 V is shown in Fig. 8, and the average photocurrent densities with standard deviation are summarized in Table 1. Under 1 sun, with the increased *n*, the photocurrent density was gradually increased from *n* = 10.5 to *n* = 20.5, and reached the maximum value at *n* = 20.5. Although the best electrochemical properties and the largest absorbance were observed at *n* = 25.5, the photocurrent density at *n* = 25.5 was lower than that at *n* = 20.5. It is possibly because much more generation of the hot electron-hole pairs and the photoexcited electron-hole pairs resulted in many unoccupied energy states, specifically holes, acting as recombination centers during electron transfer and transport. In addition, as *n* increases, the hot electrons and the photoexcited electrons can encounter much more recombination centers (e.g., holes and trap energy levels) due to a longer electron transport pathway from interfaces with Co-OEC into the back contact along the three-dimensional network of AuNPs and TNPs. Thus, the severe recombination during the longer charge transfer pathway can deteriorate the photon-to-current conversion at *n* = 25.5 despite more excellent light absorption capability and better electrochemical properties.

**Table 1:** Summary of photocurrent density values at 1.813 V vs. RHE for Co-OEC/(AuNP/TNP)_*n*_ photoanodes under various illumination conditions.

*n*	1 sun (μA/cm^2^)	UV light (μA/cm^2^)	Visible light (μA/cm^2^)	NIR light (μA/cm^2^)	Sum^*^ (μA/cm^2^)
10.5	5.03 ± 0.13	2.17 ± 0.06	2.96 ± 0.25	0.96 ± 0.18	6.09 ± 0.33
15.5	16.60 ± 0.45	4.57 ± 0.13	7.54 ± 0.13	4.39 ± 0.30	16.50 ± 0.34
20.5	54.50 ± 23.10	14.40 ± 1.99	22.40 ± 2.20	13.20 ± 1.10	50.20 ± 3.39
25.5	42.50 ± 1.19	16.60 ± 0.79	23.90 ± 1.99	12.20 ± 0.55	52.70 ± 1.94

^*^It was calculated by summing up the photocurrent densities under UV, visible, and NIR light.

However, under UV (λ < 400 nm), visible (400 nm < λ < 750 nm), and NIR light (λ > 750 nm), it was found that photocurrent density was almost constant at *n* = 20.5 and 25.5. To examine the combined effects of various spectral regions, the sum of each photocurrent density under UV, visible, and NIR light was compared with the photocurrent density under 1 sun (Table 1). A notable difference between the sum and the photocurrent density under 1 sun was observed at *n* = 25.5. The sum was lower than photocurrent density under 1 sun at *n* = 25.5. The results indicate that 1 sun illumination rather reduces the photocurrent density at *n* = 25.5. It can be explained that a lot of unoccupied states formed by photoexcitation in each spectral region cause high recombination opportunities at *n* = 25.5, inducing the loss of electrons.

On the other hand, to examine the contribution of plasmonic heating to the photocurrent density measured under NIR light, we calculated an internal photoemission (IPE) yield based on Fowler theory^60,61^. In the present study, the IPE yield was calculated in case of the emission of hot electrons from Au into TiO_2_ over the Schottky barrier (see Supplementary Information online). A ratio of the IPE yield by NIR photons to visible light photons is ~0.1 (Supplementary Table S2). In the meantime, the ratios of the photocurrent densities by NIR light to visible light were calculated (Supplementary Table S3). However, the IPE yields based on Fowler theory should be reconsidered according to previous reports^62,63^. From the reports, a calculated rate of hot electron injection was not in accord with results from Fowler theory for photon energies much larger than a Schottky barrier height because assumptions used in Fowler theory conflict with real experiments^62–64^. If the Schottky barrier height is 1 eV, the results from Fowler theory is overestimated above 2 eV of photon energies compared to the rate of the injection^62,63^. Thus, in case of the emission of hot electrons from Au into TiO_2_, it can be said that the IPE yields in the NIR region are well approximated and IPE yields in the visible region are overestimated, resulting in the ratio of the yield smaller than 0.1. As a result, the ratios of the photocurrent densities are much larger than the ratio of the IPE yield, which indicates that plasmonic heating could contribute to the photocurrent density in addition to the hot electron injection under NIR light.

**Figure 9:**
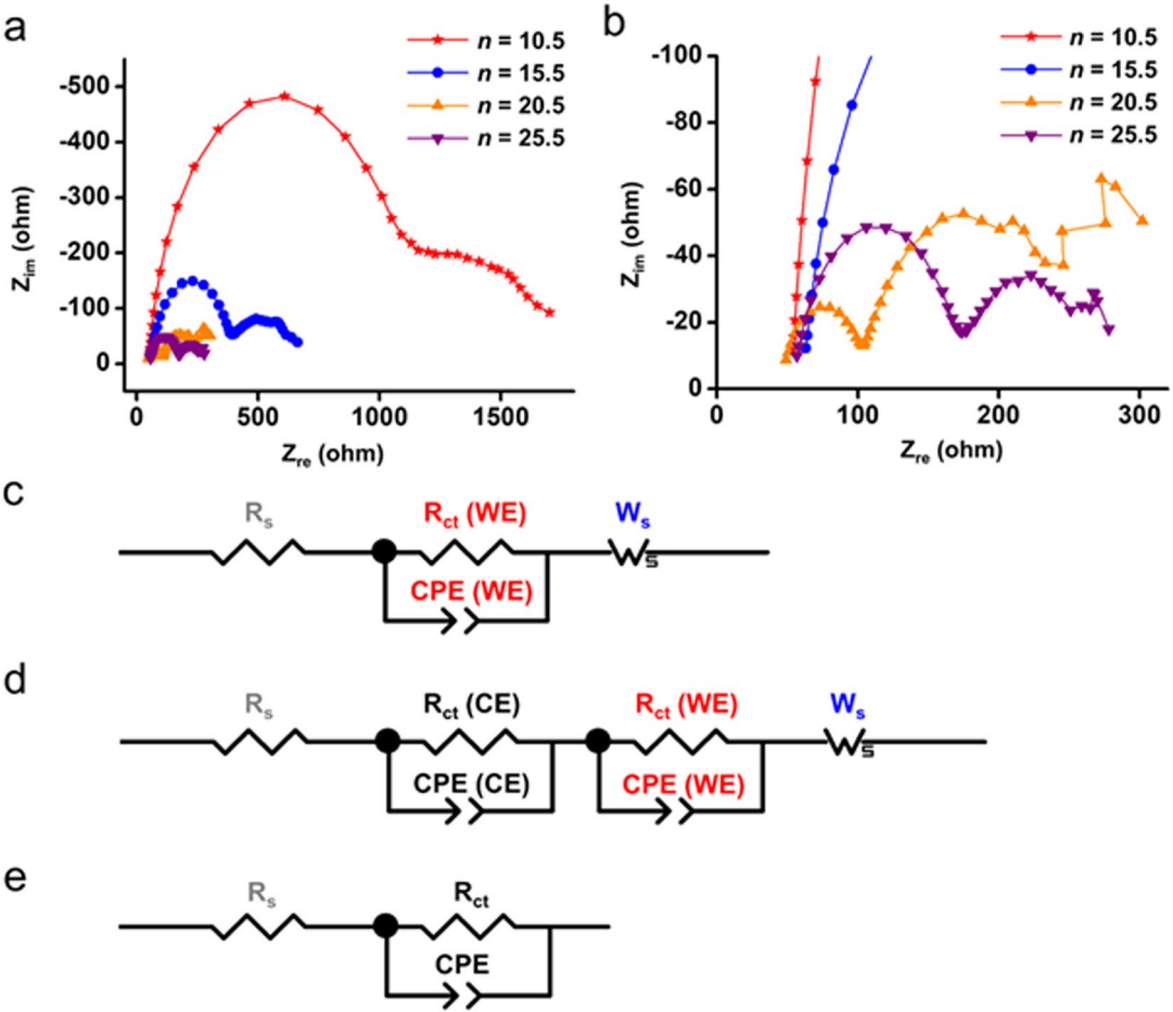
Representative Nyqiust plots in the dark (**a**), their magnified plots (**b**), EC model for *n* = 10.5, 15.5, and 25.5 (**c**), EC model for *n* = 20.5 (**d**), and Randles electrical circuit (**e**) at 1.813 V vs. RHE.

Electrochemical impedance spectroscopy (EIS) was also used to identify the electrical components affected by irradiation and their contribution to the photocurrent density. Impedance was measured three times at 1.813 V, where oxygen evolution reaction occurs, both under 1 sun and in the dark to obtain more exact values and rule out effects by transient changes in the surrounding environment because water oxidation is a heterogeneous reaction where the phases of reactants and products are different (Supplementary Fig. S7). Representative Nyquist plots in the dark at 1.813 V are shown in Fig. 9a,b. Nyquist plots for *n* = 10.5, 15.5, and 25.5 showed a large semicircle in the high-frequency region and a distorted arc in the low-frequency region, but the Nyquist plot for *n* = 20.5 has a smaller semicircle in the high-frequency region, a bigger semicircle in the middle frequency region, and a distorted arc in the low-frequency region. Thus, two kinds of equivalent circuit (EC) models were assumed to fit the impedance data: one for *n* = 10.5, 15.5, and 25.5 and the other for *n* = 20.5 (Fig. 9c,d). The EC models were assumed considering that the Randles electrical circuit indicates one semicircle in the Nyquist plot (Fig. 9e). The EC model used to fit the impedance data for the photoanodes consists of some electrical components such as a resistance and a constant phase element (CPE). Series resistance, R_s_, includes sheet resistance of the substrate, electrical wiring, and solution resistance. In addition, the EC model contains charge transfer resistance (R_ct_ (CE)) and capacitance (CPE (CE)) at interfaces of the counter electrode/electrolyte. Finally, charge transfer resistance (R_ct_ (WE)) and capacitance (CPE (WE)) at the interface of the working electrode/electrolyte are assigned to the bigger semicircle in low-frequency region. Note that CPE is the generalized concept of conventional capacitances for EC modeling^62^. The distorted arcs are attributed to diffusion impedance in the low-frequency region, reflected in the EC model as Warburg short impedance (W_s_)^65^.

Fitting the results using the EC models is summarized in Table 2. Irradiation caused a notable decrease in R_ct_ (WE) and W_s_, but other electrical components were very similar. The decrease in R_ct_ (WE) is attributed to charge generation by irradiation, specifically the hot electrons in AuNPs and the photoexcited electrons in TNPs and TiO_2_ thin film. When the hot electrons and the photoexcited electrons are generated, energetic holes are simultaneously generated and can act as acceptors for electrons transferred from electrolytes. Besides, plasmonic heating can accelerate the reaction rate of Co-OEC, resulting in rapidly scavenging the energetic holes from AuNPs and TiO_2_. Thus, the existence of the energetic holes and plasmonic heating facilitates charge transfer at the photoanode/electrolyte interfaces, causing R_ct_ (WE) drop. On the other hand, the decreased W_s_ is attributed to plasmonic heating by AuNPs and enhanced mass transfer by the increased concentration gradient. A large number of the hot electrons cannot be injected to TiO_2_ over the Schottky barrier due to their insufficient energy and distance from the interfaces of Au/TiO_2_. They are eventually damped and converted to thermal energy via electron-electron and electron-phonon relaxations^25^. Thermal energy by plasmonic heating is transferred to surrounding environment, specifically Co-OEC. The localized heat could increase the proton transfer within Co-OEC through multiple hopping steps. Fast generation of the protons due to R_ct_ (WE) drop can increase the concentration gradient of protons, facilitating proton transfer between the photoanode and counter electrode via a diffusion process.

**Table 2:** Summary of the results obtained by fitting the Nyquist plots with the corresponding EC model at 1.813 V vs. RHE for Co-OEC/(AuNP/TNP)_*n*_ photoanodes under 1 sun and in the dark.

Electrical components	*n* = 10.5		*n* = 15.5		*n* = 20.5		*n* = 25.5	
	dark	1 sun	dark	1 sun	dark	1 sun	dark	1 sun
R_s_	47.05	47.07	53.42	47.91	46.50	45.74	51.10	50.63
CPE (CE) (×10^5^)					2.40	2.34		
R_ct_^(CE)^					54.04	54.49		
CPE ^(WE)^ (×10^5^)	1.25	1.27	1.05	1.07	0.00292	0.00283	2.00	2.07
R_ct_^(WE)^	990.90	974.70	274.90	265.83	121.50	105.30	95.18	87.34
W_s_	636.77	588.83	354.93	323.27	127.95	105.80	147.17	134.47

## Conclusions

In this work, we have demonstrated that a plasmonic photoanode based on the multilayered heterostructures with water oxidation catalysts can have excellent light absorption capability and photoactivity in all the spectral regions for PEC water splitting. The multilayered heterostructure enables efficient separation and collection of hot carriers by increasing the interfacial area of Au/TiO_2_. Large amounts of AuNPs and TNPs via LbL self-assembly can enhance light absorption capability and LSPR effect. In addition, the incorporation of Co-OEC can assist charge separation between energetic electrons and holes by scavenging the holes. To demonstrate PEC performances of the photoanodes, we conducted PEC analysis under various condition. The photoanodes showed the photopotential, resulting in the photocurrent density under various illumination conditions. The photocurrent density under various illumination conditions indicated the existence of the hot electron injection from AuNPs into TNPs and TiO_2_ thin film and the photoexcited electron-hole pairs in TiO_2_. The photoactivity of the photoanodes was influenced by charge transport within the multilayered heterostructures, so photocurrent density was determined by the degree of charge recombination, as shown in the comparison between *n* = 20.5 and 25.5. Impedance analysis confirmed that generation of energetic electron-hole pairs could assist PEC water splitting under 1 sun illumination. The present study suggests a simple method to design an efficient plasmonic photoelectrode for photocatalytic and PEC reactions.

## Experimental Section

### Chemicals

Gold (III) chloride trihydrate (HAuCl_4_∙3H_2_O), cysteamine hydrochloride, NaBH_4_, TBAH, TTIP (Ti[OCH(CH_3_)_2_]_4_), cobalt (II) nitrate hexahydrate (Co(NO_3_)_2_∙6H_2_O), potassium phosphate monobasic, and potassium phosphate dibasic trihydrate were purchased from Sigma-Aldrich (St. Louis, MO, USA). 2-Propanol and isopropanol were obtained from Samchun Pure Chemical Co., Ltd (Pyeongtaek-si, Republic of Korea). ITO-coated glasses (sheet resistance = 10 mΩ cm^−2^, 10 mm× 37 mm × 0.7 mm Th) were purchased from Taewon Scientific Co., Ltd (Seoul, Republic of Korea).

### Synthesis of Positively Charged AuNPs

Positively charged AuNPs were prepared by reducing gold salts with cysteamine as a capping agent according to a previously reported method^66,67^. Forty milliliters of 1.42 mM HAuCl_4_ in deionized water were mixed with 400 μL of 213 mM cysteamine hydrochloride in deionized water in a glass vial at room temperature. The mixture was magnetically stirred at 200 rpm for 20 min at room temperature in the dark. Fresh 1 mL of 0.1 mM NaBH_4_ in deionized water was prepared and then quickly added to the mixture solution of HAuCl_4_ and cysteamine. The mixture was magnetically stirred at 200 rpm for 60 min at room temperature in the dark. The synthesized AuNPs were dialyzed using a Spectra/Por 1 Dialysis membrane (molecular weight cut-off = 6–8 kDa) with magnetic stirring for 24 h at room temperature in the dark. The nanoparticles were stored in a glass bottle in a refrigerator (4 °C) in the dark until use.

### Synthesis of Negatively Charged TNPs

Negatively charged TNPs were prepared using a non-aqueous sol-gel method using TTIP and TBAH as a precursor and a base, respectively. Deionized water (300 mL) in a 1 L round flask was heated in an oil bath at 100 °C for 60 min. Forty-six milliliters of 27.2 mM TBAH in 2-propanol and 10 mL of 337.8 mM TTIP in 2-propanol were prepared in a conical tube using vortex mixing, respectively. The TTIP solution was added to a fresh 1 L round flask. The TBAH solution was slowly added to the TTIP solution dropwise with magnetic stirring at 500 rpm at room temperature, and then the pre-boiled deionized water (224 mL) was slowly added to the mixture. The final mixture was refluxed at 100 °C for 48 h with magnetic stirring at 500 rpm. The synthesized TNPs were purified using a Centricon (Millipore Amicon Ultra-15, nominal molecular weight limit = 100 kDa). Six Centricon tubes containing the TNPs were put into a swinging bucket rotor centrifuge, and centrifugal filtering was conducted at 2500 rcf for 30 min at room temperature. This procedure was repeated four times. The TNPs of final filtrate were suspended in deionized water.

### Fabrication of (AuNP/TNP)_*n*_ Multilayered Heterostructures

ITO-coated glasses of 10 mm × 17.5 mm × 0.7 mm Th were prepared by dicing. The ITO-coated glasses were cleaned using ultrasonic cleaning with acetone, ethanol, and deionized water, then dried using a blower. The ITO-coated glasses were taped using a polyimide tape saving active area 1 cm^2^. The ITO-coated glasses were cleaned using UV/Ozone cleaning. A TiO_2_ thin film of 10 nm was deposited onto the ITO-coated glasses using an electron-beam evaporator (SNTEK VER-5004) with an accelerating voltage of 7.2 kV at a current rate of 0.5 Å s^−1^. A surface of TiO_2_/ITO substrates was treated with oxygen plasma. The substrates were dipped into a colloidal solution of AuNPs for 60 min in a petri dish under twist shaking, and then the substrates were washed with deionized water and dried using a blower. The substrates were dipped into a colloidal solution of TNPs in the same manner and then washed and dried in the same manner. This procedure was repeated, resulting in (AuNP/TNP)_*n*_ multilayered heterostructures. Finally, a layer of AuNPs was coated as a top layer.

### Electrodeposition of Co-OEC

Electrodeposition of Co-OEC was performed in a two-compartment cell (H cell) containing 100 mL of 0.1 M potassium phosphate buffer (pH 7) in a side and 100 mL of 0.1 M potassium phosphate buffer (pH 7) and 0.5 mM Co(NO_3_)_2_ in the other side at room temperature using electrochemical system (Ivium-n-Stat Multichannel electrochemical analyzer and IviumSoft) and conducted using three-electrode system. (AuNP/TNP)_*n*_ multilayered heterostructures were used as the WE. Ag/AgCl and a platinum wire were used as the RE and CE, respectively. A potential of 1.513 V was applied until an amount of flowed charges was 30 mC.

### Characterizations

A zeta potential analyzer (Otsuka Electronics ELSZ-1000) was used to analyze a surface charge of AuNPs and TNPs in deionized water. A UV-vis spectrometer (SHIMADZU UV-1800) was used for measuring light absorption of AnNPs, TNPs, and (AuNP/TNP)_*n*_ multilayered heterostructures. Field emission TEMs (FEI Company Tecnai F20 and FEI Company Talos F200X) were used with an accelerating voltage of 200 kV for analyzing a size and shape of AuNPs and TNPs. Surface images of SEM were obtained by field emission SEMs (FEI Company Nova230 and Hitachi SU5000) with an accelerating voltage of 5 kV for (AuNP/TNP)_*n*_ multilayered heterostructures and field emission SEMs (Hitachi SU8230 and Hitachi SU5000) with an accelerating voltage of 5 kV for Co-OEC/(AuNP/TNP)_*n*_ photoanodes, respectively. EDAX spectra were recorded using a field emission SEM (Hitachi SU8230) with an accelerating voltage of 15 kV for elemental analysis of (AuNP/TNP)_*n*_ multilayered heterostructures and Co-OEC/(AuNP/TNP)_*n*_ photoanodes.

### PEC Analysis

All PEC measurements were performed in a quartz cell containing 100 mL of 0.1 M potassium phosphate buffer (KH_2_PO_4_/K_2_HPO_4_) at pH 7 using electrochemical system (Ivium-n-Stat Multichannel electrochemical analyzer and IviumSoft) and conducted using a three-electrode system at room temperature. Co-OEC/(AuNP/TNP)_*n*_ photoanodes were used as the WE. Ag/AgCl and a platinum mesh were used as the RE and CE, respectively. Potentials were measured against the Ag/AgCl and converted to values against RHE using the following equation: E (vs. RHE) = E (vs. Ag/AgCl) + 0.197 V + 0.059 × pH. In addition, 1 sun illumination was applied to the photoanodes using a solar simulator (ASAHI SPECTRA HAL-320), and various light conditions were applied using 1 sun illumination with optical filters in all PEC measurements. The photoanodes were illuminated at a side of the ITO-coated glass as backside illumination. LSV was carried out at a scan rate of 2 mV s^−1^ with a scan range of 0.413 V to 2.013 V. Tafel plots were obtained from LSV curves considering Tafel equation: η = β log *J* + α, where η is an overpotential, *J* is a current density, and β is a Tafel slope. Photocurrent densities were measured by CA under chopped illumination, and the light was switched on and off at an interval of 5 min at 1.813 V. EIS was conducted at 1.813 V with 0.01 V amplitude and 100000-0.01 Hz of the frequency range.

## Electronic supplementary material

Supplementary information
