# Supplementary material for: Multilayered Plasmonic Heterostructure of Gold and Titania Nanoparticles for Solar Fuel Production
Source: Sci Rep. 2018 Jul 11;8:10464. doi: 10.1038/s41598-018-28789-w (PMC6041279; doi:10.1038/s41598-018-28789-w)
Supplement: Supplementary file 1 — Supplementary information [file 41598_2018_28789_MOESM1_ESM.pdf]

Supplementary Information

Multilayered Plasmonic Heterostructure of Gold and Titania Nanoparticles for Solar Fuel Production

Jeonga Kim[1], Ho Yeon Son[1], and Yoon Sung Nam[1,2,*]

[1]Department of Materials Science and Engineering and [2]KAIST Institute for the NanoCentury, Korea Advanced Institute of Science and Technology, 291 Daehak-ro, Yuseong-gu, Daejeon, 34141, Republic of Korea

*Address correspondence to yoonsung@kaist.ac.kr

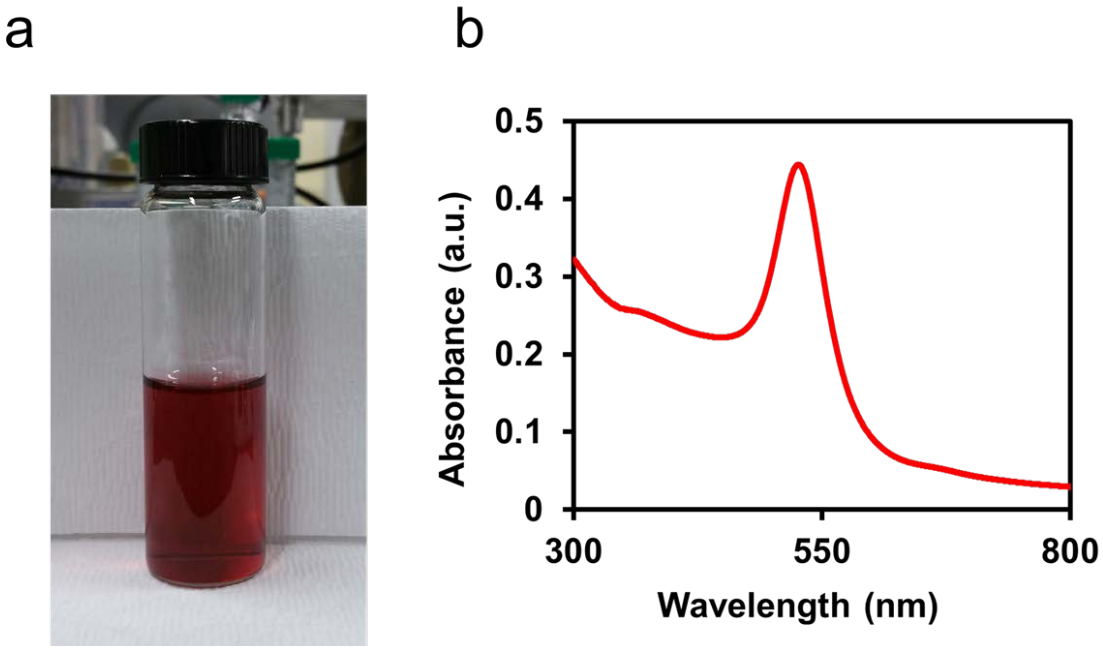

Supplementary figure S1. Digital photograph (a) and absorption spectrum (b) of the synthesized AuNPs. A peak is observed at 526 nm.

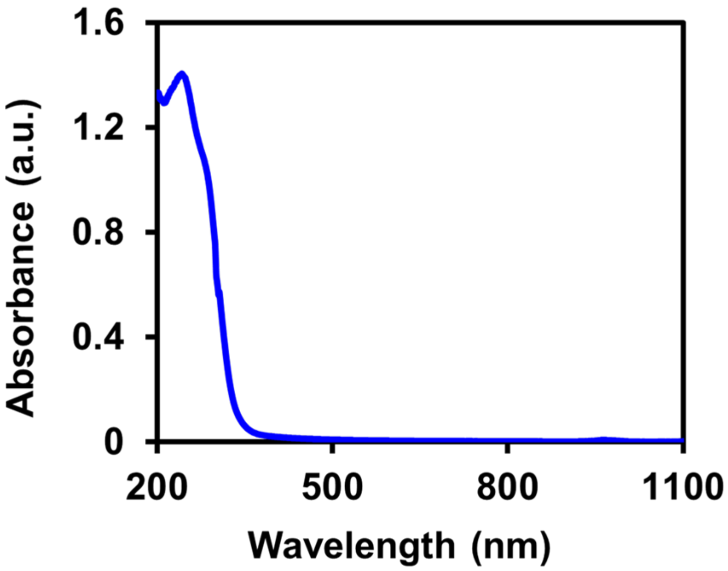

Supplementary figure S2. Absorption spectrum of the synthesized TNPs.

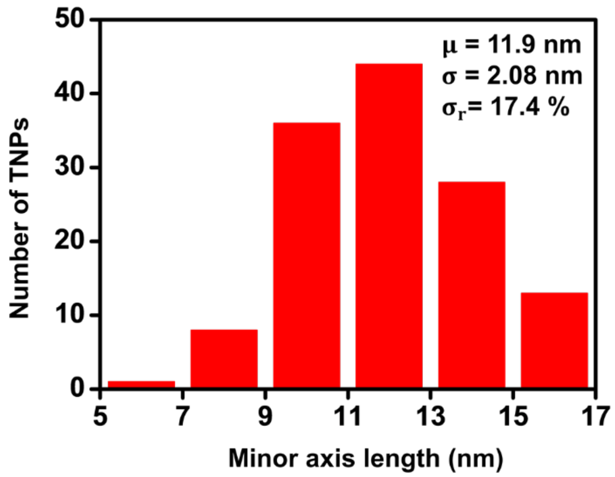

Supplementary figure S3. Particle size distribution histogram for the minor axis length of the synthesized TNPs.

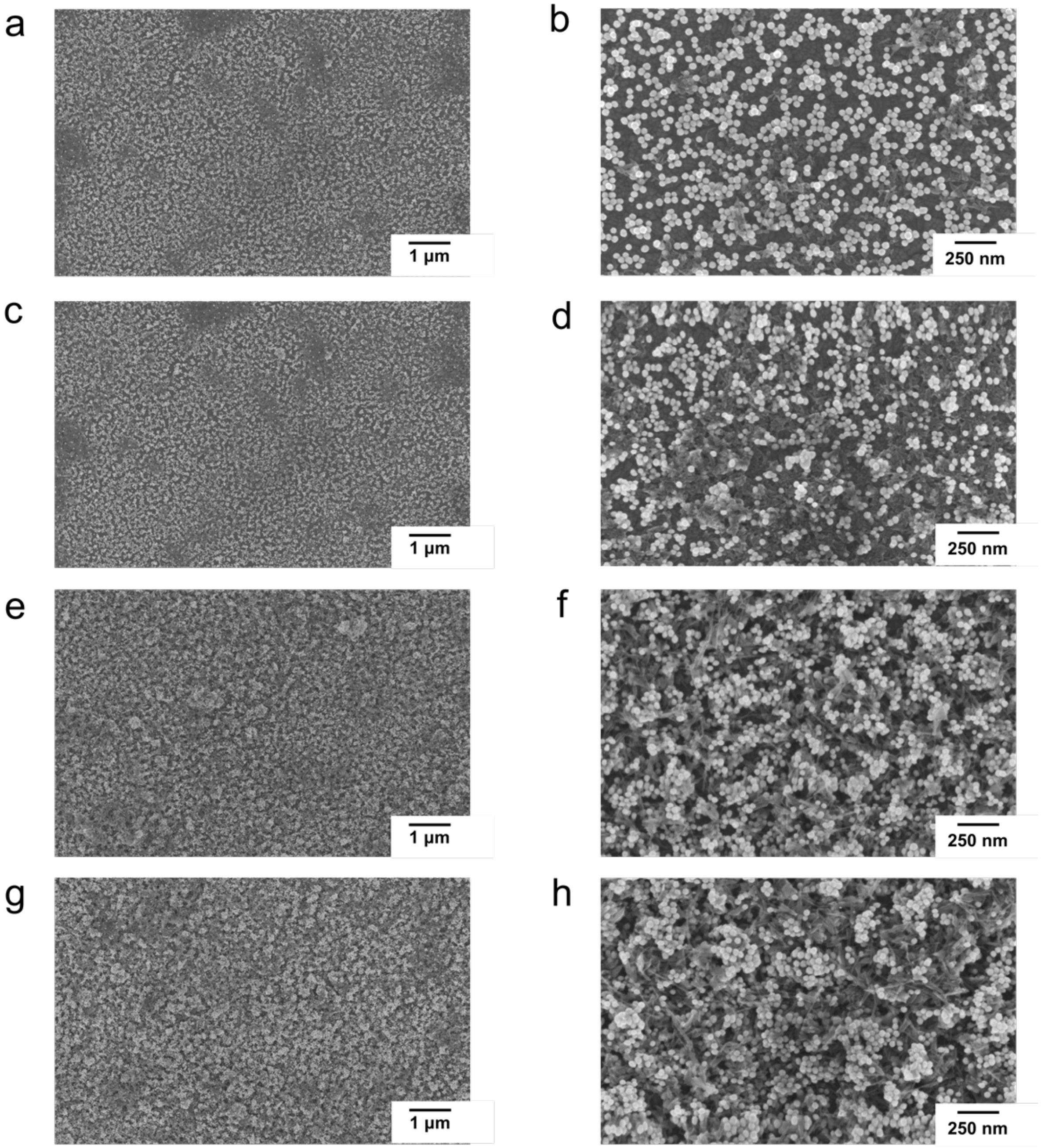

Supplementary figure S4. SEM images of $(AuNP/TNP)_n$ multilayered heterostructures: n = 10.5 (a and b), 15.5 (c and d), 20.5 (e and f), and 25.5 (g and h).

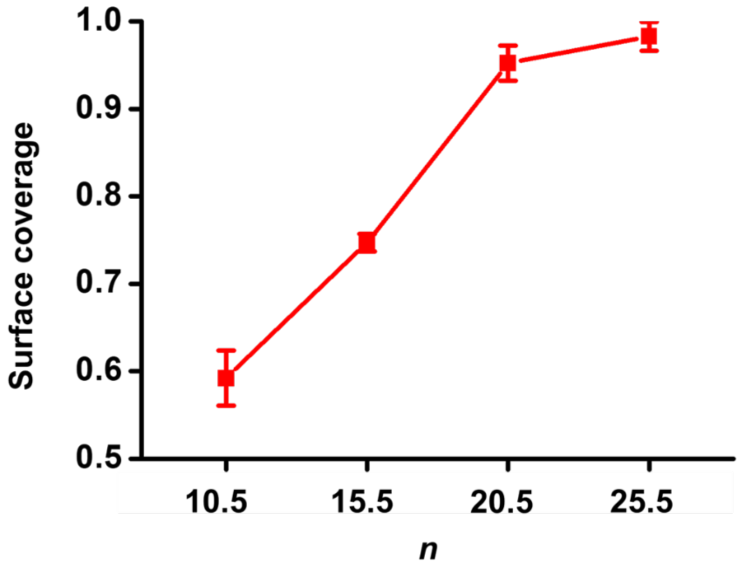

Supplementary figure S5. Surface coverage by NPs calculated by using SEM image thresholding of $(AuNP/TNP)_n$ multilayered heterostructures for n = 10.5-25.5.

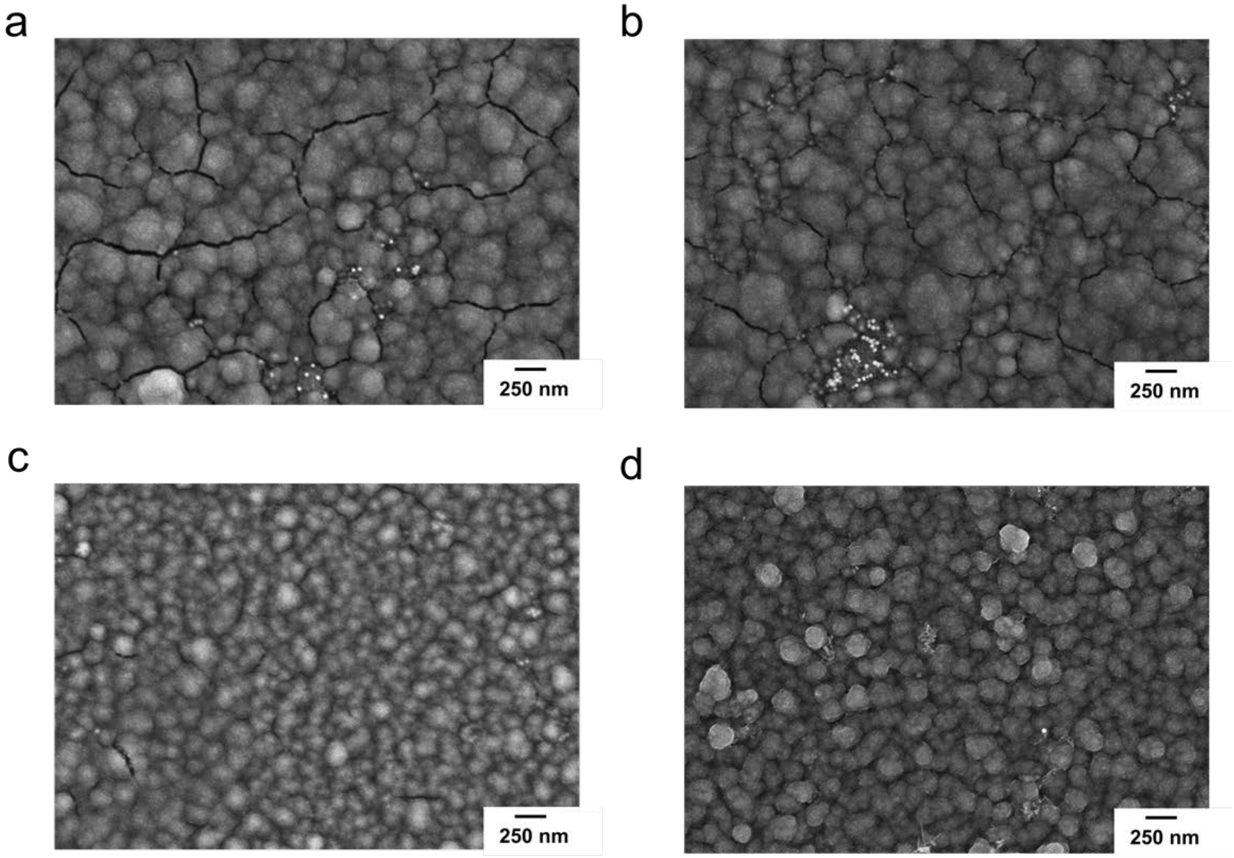

Supplementary figure S6. SEM images of Co-OEC/(AuNP/TNP)$_n$ photoanodes: n = 10.5 (a), 15.5 (b), 20.5 (c), and 25.5 (d).

Supplementary table S1. The number of Co-OEC/(AuNP/TNP)$_n$ samples used for obtaining LSV curves at each *n*.

n	# of samples
10.5	5
15.5	4
20.5	6
25.5	2

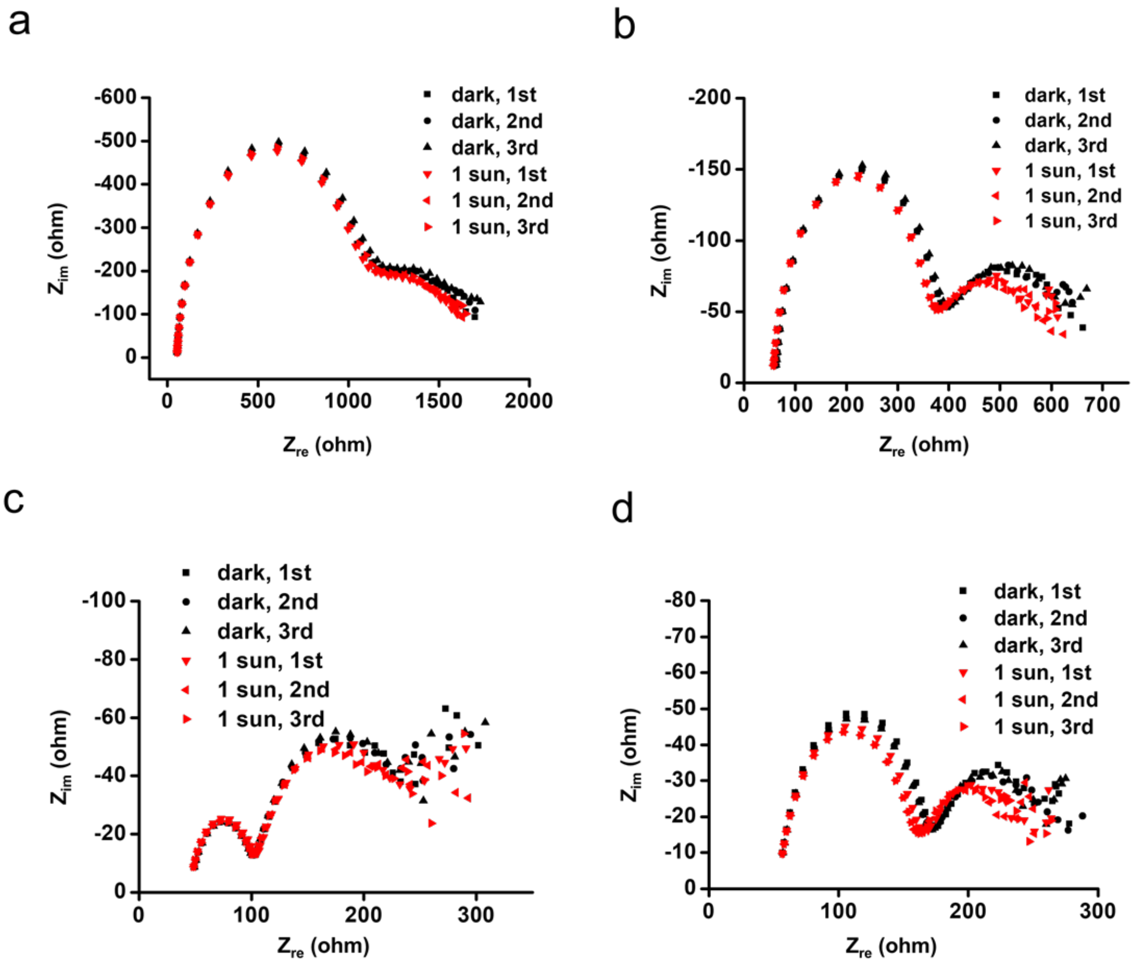

Supplementary figure S7. Nyqiust plots at 1.813 V vs. RHE in the dark and under 1 sun illumination for Co-OEC/(AuNP/TNP)$_n$ photoanodes: n = 10.5 (a), 15.5 (b), 20.5 (c), and 25.5 (d).

Internal photoemission (IPE) yield

According to Leenheer et al., simple IPE yield based on Fowler theory was utilized in emission of hot electrons from a metallic nanostructure into a semiconductor over the Schottky barrier and expressed as follows,

$$Y \approx \frac{1}{8E_F}\frac{(\hbar\omega - \phi_b)^2}{\hbar\omega}$$

where $\hbar$ is the reduced Plank constant, ω is the angular frequency of light, ϕ_b is the Schottky barrier height, and E_F is the Fermi energy of the metal (Supplementary Fig. S8)[1]. In the present study, the metal and semiconductor were regarded as Au and TiO_2, respectively. Thus, E_F and ϕ_b were assumed to be 5.5 eV and 1.1 eV, respectively[1-2]. The IPE yields in the visible light and NIR regions were obtained by summing up the yields at all wavelengths, and a ratio of the yield by NIR light to visible light was calculated (Supplementary Table S2). The ratio of the yield was compared with calculated ratios of average photocurrent densities by NIR light to visible light (Supplementary Table S3).

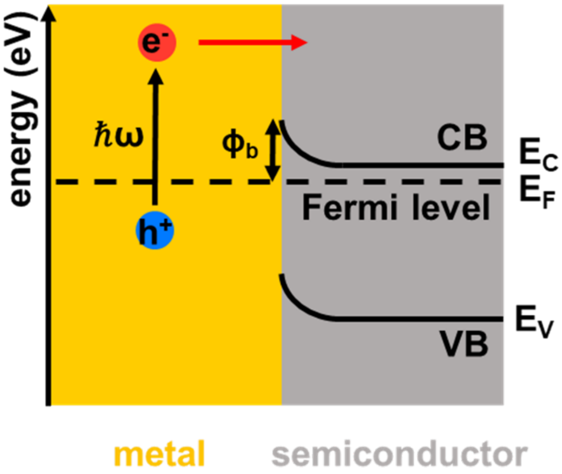

Supplementary figure S8. Band diagram for IPE of hot electrons from a metal to a semiconductor over the Schottky barrier.

Supplementary table S2. IPE yield of hot electrons from Au to TiO_2 over the Schottky barrier in the visible light and NIR region.

Spectral regions	IPE yields
Visible light region	4.42
NIR region	0.41

Supplementary table S3. Ratios of average photocurrent densities by NIR light to visible light.

n	Ratios
10.5	0.32
15.5	0.58
20.5	0.59
25.5	0.51

References

(1) Leenheer, A. J., Narang, P., Lewis, N. S. & Atwater, H. A. Solar energy conversion via hot electron internal photoemission in metallic nanostructures: efficiency estimates. *J. Appl. Phys.* **115**, 134301 (2014).

(2) Lee, J., Mubeen, S., Ji, X., Stucky, G. D. & Moskovits, M. Plasmonic photoanodes for solar water splitting with visible light. *Nano Lett.* **12**, 5014−5019 (2012).
